# Supplementary material for: MetaRibo-Seq measures translation in microbiomes
Source: Nat Commun. 2020 Jun 29;11:3268. doi: 10.1038/s41467-020-17081-z (PMC7324362; doi:10.1038/s41467-020-17081-z)
Supplement: Supplementary file 10 — Supplementary Data 7 [file 41467_2020_17081_MOESM10_ESM.zip › File2/Confidence_High_Taxonomy/227599_out.krona.html]

Javascript must be enabled to view this page.

members
magnitude
magnitudeUnassigned
count
unassigned
taxon
rank

227599\_out

17

superkingdom
17
2

phylum
17
1239

186801
17
class

order

SRS142503\_contig\_number\_11790
1
17
186802

541000
family
5

1263
5
genus

657323

SRS023715\_contig\_number\_27223SRS1041130\_contig\_number\_contig-100\_308.30101
2
species

2
species

SRS019068\_contig\_number\_4468SRS049773\_contig\_number\_15375
2293151

species
1

SRS017103\_contig\_number\_36505
2293236

11
family
186803

189330
genus
1

species
1
88431

SRS147022\_contig\_number\_contig-100\_9511.9511

genus
10
572511

6
species

SRS015217\_contig\_number\_29386SRS015431\_contig\_number\_55473SRS019445\_contig\_number\_contig-100\_461.185149SRS023914\_contig\_number\_22032SRS1041091\_contig\_number\_18944SRS143991\_contig\_number\_16188
765821


SRS042284\_contig\_number\_5494SRS077502\_contig\_number\_18605SRS148721\_contig\_number\_12456
1737424
3
species

1520805

SRS077454\_contig\_number\_12940
1
species
